# Mir-24-3p downregulation contributes to VP16–DDP resistance in small-cell lung cancer by targeting *ATG4A*

**DOI:** 10.18632/oncotarget.2787

**Published:** 2014-11-16

**Authors:** Banzhou Pan, Yitian Chen, Haizhu Song, Yichen Xu, Rui Wang, Longbang Chen

**Affiliations:** ^1^ Department of Medical Oncology, Jinling Hospital, School of Medicine, Nanjing University, Nanjing, China

**Keywords:** miR-24-3p, autophagy, chemoresistance, small-cell lung cancer, mechanism, *ATG4A*

## Abstract

Although the combination of etoposide (VP16) and cisplatin (DDP) is widely used as a first-line treatment for advanced-stage small-cell lung cancer (SCLC), chemoresistance limits its clinical use. Abnormalities of autophagy are associated with tumor chemoresistance. The present study found that miR-24-3p, a recently discovered microRNA, is significantly downregulated in VP16–DDP-resistant SCLC cells (H446/EP) compared with VP16–DDP-sensitive parent cells (H446). Forced expression of miR-24-3p sensitized H446/EP cells to VP16–DDP treatment because of a blockade of autophagic activity. We further found that downregulated miR-24-3p enhanced autophagy activation as it directly targets and inhibits autophagy-associated gene 4A (*ATG4A*). Overexpression of miR-24-3p into H446/EP cells led to reduction of the ATG4A protein level, allowing SCLC cells to resensitize to VP16–DDP. We conclude that miR-24-3p regulates autophagy by targeting ATG4A. Inhibition of autophagy by increasing miR-24-3p could be the basis of a strategy to prevent and treat SCLC with combination chemotherapy, particularly in chemoresistant disease.

## INTRODUCTION

Small-cell lung cancer (SCLC) constitutes approximately 15% of all lung cancers and is one of the most common malignant tumors worldwide [1, 2]. The combination of etoposide (VP-16) and cisplatin (DDP) (VP16–DDP) is the most widely used systemic therapy for SCLC, especially for advanced-stage disease [3]. SCLC is characterized by an aggressive propensity for early dissemination and rapid development of chemoresistance during the treatment, although patients initially show high response rates [4]. Patients with resistant disease suffer early relapse; overall survival at 5 years is less than 5% [4]. Hence, SCLC chemoresistance is the major obstacle in clinical application of chemotherapy.

MicroRNAs (miRNAs) are small non-coding RNAs that modulate gene expression at the post-transcriptional level. They bind to 3′-untranslated regions (3′-UTR) of target mRNAs, leading to mRNA destabilization and translational repression [5]. Although specific miRNA subsets are linked to various basic cellular processes, such as cell proliferation, differentiation and apoptosis [6, 7], the role of miRNA in modulating autophagy remains a promising area of research. Specific miRNAs are differentially expressed between normal and cancer cells [8], and modulate cancer therapy response and resistance [9]. Nevertheless, miRNA expression in chemoresistant SCLC is not widely investigated, and the mechanisms that underlie aberrant miRNAs expression are not well understood.

Autophagy is an evolutionarily conserved intracellular catabolic process by which cells enable removal of damaged cellular components to improve energy production under diverse stressful conditions [10]. This process is characterized by the formation of autophagosomes—double-membrane vesicles that engulf portions of cytoplasm and then fuse with lysosomes for degradation [10]. At least 36 autophagy (*ATG*) genes are primarily involved in the autophagy process in mammalian cells [11]. An indispensable step of autophagosome formation is the proteolytic cleavage of microtubule-associated protein 1 light chain 3 (LC3) to generate LC3-I isoform with an exposed C-terminal glycine residue, which enables the conjugation with phophatidyl-ethanolamine to yield LC3-II. Proteolytic cleavage is also responsible for the deconjugation of LC3-II to LC3-I for LC3 recycling [12]. These two crucial events are catalyzed by the cysteine protease ATG4 that acts as both the conjugating and deconjugating enzyme [13, 14]. To date, 4 Atg4 paralogs (ATG4A, ATG4B, ATG4C and ATG4D) have been reported in mammals, with substrate spectra for different LC3 forms and homologs [15]. Autophagic activity is attenuated in *ATG4B*- or *ATG4C*-knockout mice [16, 17]. However, the role of ATG4A in autophagy and the dependence of ATG4-mediated autophagy on cancer cell proliferation remain unclear.

The exact role of autophagy during tumor therapy is elusive, as autophagy can be a pro-survival mechanism to deteriorate therapeutic outcomes or act as programmed cell death to improve overall anti-tumor efficacy [18]. Although understanding of the mechanisms of autophagy has substantially advanced, information on the regulation of this complex process is limited. In this study, we focused on determining the role of miRNAs in the development of VP16–DDP resistance in SCLC related to autophagy. Following miRNA array analysis, we screened dysregulated miRNAs with expression levels that differed by ≥ 250% either way between VP16–DDP-sensitive (H446) and VP16–DDP-resistant (H446/EP) SCLC cells. We found that miR-24-3p, a down-regulated miRNA in H446/EP cells, had a predominant depressing effect on autophagy activation, and enhanced the cytotoxicity of VP16–DDP treatment.

Furthermore, to our knowledge, our study is the first to confirm *ATG4A* as a direct functional target of miR-24-3p in SCLC. Whereas treatment with VP16–DDP for SCLC promotes autophagy (which facilitates apoptosis and cell death), overexpressed miR-24-3p results in downregulated ATG4A and decreases autophagy. Our results indicate a potential therapeutic target for reinforcing the efficacy of VP16–DDP chemotherapy.

## RESULTS

### Differential miRNA expression in VP16–DDP-sensitive and -resistant SCLC cells

**Figure 1:**
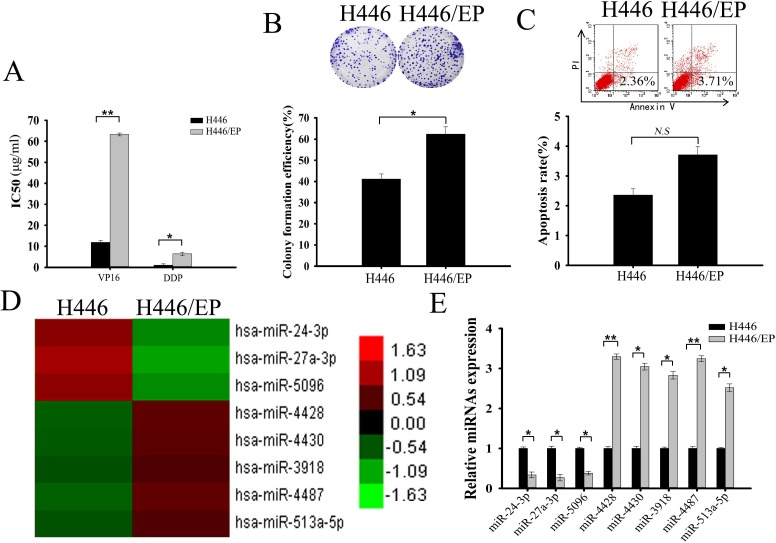
Differential miRNA expression profile of VP16–DDP-sensitive and -resistant H446 cells (A) MTT assay showed H446/EP cells to be much more resistant to combined VP16–DDP therapy than H446 cells. (B) Colony formation assay showed significantly enhanced proliferating ability of H446/EP cells *in vitro*. (C) Flow cytometric analysis showed H446 cell apoptotic rate is statistically similar to that of H446/EP cells. (D) Heat map of 8 miRNAs that (increased or decreased to in expression at least 2.5 fold) in H446/EP cells relative to their expression in parental H446 cells (columns: cell lines; rows: probe sets). Heat map indicates high (red) or low (green) expression relative to mean (as shown in the scale). (E) Real-time quantitative RT-PCR (qRT-PCR) shows relative expression of 8 dysregulated miRNAs in H446 and H446/EP cells (control: U6). Results are from ≥ 3 independent experiments, expressed as mean ± SD.*P < 0.05; **P < 0.01, *N.S, P>0.05*.

We established VP16–DDP-resistant SCLC cells (H446/EP) from VP16–DDP-sensitive cells (H446) by continuous exposure to VP16 and DDP. A MTT assay measured sensitivity of both cell types to these two cytotoxic agents. The IC_50_ values for VP16 were 11.89μg/ml and 63.27μg/ml in H446 and H446/EP, respectively; and for DDP were 1.02μg/ml and 6.38μg/ml, respectively (Fig. 1A). A colony formation assay showed significantly enhanced proliferating ability of H446/EP cells (Fig. 1B). However, flow cytometry showed minimal change in apoptosis for H446/EP cells compared with H446 cells (Fig. 1C).

MicroRNA array results found 8 miRNAs that were expressed by ≥250% in either H446 or H446/EP cells compared with the other (Fig. 1D; Supplementary Table 1). Real-time quantitative RT-PCR (qRT-PCR) affirmed that 3 miRNAs were downregulated, and 5 were upregulated, in H446 cells, in accordance with the microarray data (Fig. 1E).

### VP16–DDP-resistant SCLC cells exhibited increased autophagy

**Figure 2:**
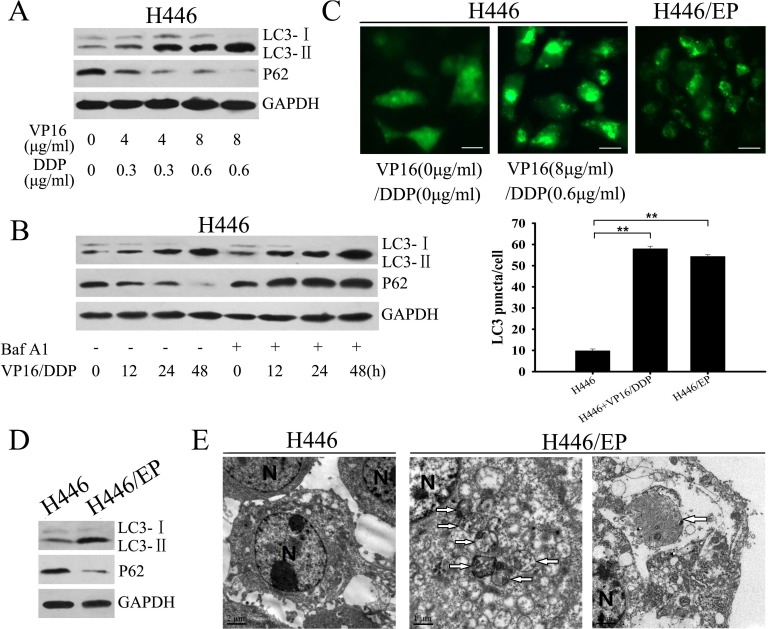
H446/EP cells exhibited heightened autophagy (A) H446 cells were treated with the indicated concentrations of VP16 and DDP for 48 h. Whole cell lysates were analyzed by western blot using LC3 and P62 antibodies (control: GAPDH). (B) H446 cells pretreated with or without Bafilomycin A1 (Baf A1, 20nM, 2h) were exposed to 8 μg/ml VP16 and 0.6 μg/ml DDP (VP16–DDP) for varying periods. Whole cell lysates were analyzed by western blot. (C) Parental H446 cells and VP16–DDP-resistant H446/EP cells were transiently transfected with a GFP-LC3 construct; 24 hours later, parental cells were exposed to VP16–DDP for another 24 h. GFP-LC3 dot formation was analyzed as described in Materials and Methods (mean ±SD of 3 independent experiments; **P<0.01; bar: 50 μm). (D) Western blot for LC3, P62 and GAPDH expression in H446 cells and H446/EP cells. (E) Transmission electron micrographs of H446 cells and H446/EP cells showing characteristic autophagosomes. Some of these double membrane-surrounded autophagosomes contained remnants of organelles, including mitochondria. The right far figure showed a characteristic autophagosome in the elongation stage. N: nucleus. Results are presented as mean ± SD of values obtained in three independent experiments. *P<0.05; **P < 0.01.

Autophagy reportedly occurs in response to chemotherapy and plays a major role in development of chemoresistance in tumor cells [19]. Western blot analysis showed that in H446 cells, VP16–DDP treatment led to a dose- and time-dependent increase in the LC3-II/LC3-I ratio, and decreased P62 levels, two selective markers of autophagy (Fig. 2A, B). Autophagy flux was assessed by detecting levels of LC3-II protein in the presence or absence of bafilomycin A1 (Baf A1). As an autophagy-lysosomal inhibitor, Baf A1 promotes accumulation of autophagic vacuoles by blocking fusion of autophagosomes with lysosomes, thus preventing LC3-II degradation by acidic organelles [20]. We found that adding Baf A1 further increased LC3-II levels in H446 cells compared with drug therapy alone (Fig. 2B). The effect of VP16–DDP on autophagy was confirmed by a GFP-LC3 punctate formation assay; after VP16–DDP treatment, H446 cells that express GFP-LC3 showed GFP-LC3 signals shifting from a diffuse pattern to a punctuate pattern, which reflects conversion of cytoplasmic LC3-I to the -associated autophagosome form, LC3-II (Fig. 2C). We next assessed autophagy activity in VP16–DDP-resistant cell line H446/EP, which had been established in our lab. Western blot analysis showed baseline LC3-II levels to be higher in H446/EP cells than in their H446 parental cells (Fig. 2D). Elevated autophagy activity in H446/EP was affirmed by transmission electron microscopy and GFP-LC3 fluorescence microscopy, measured as characteristic autophagosomes formation and an increased percentage of punctate GFP^+^ cells, respectively (Fig. 2E, C).

**Figure 3:**
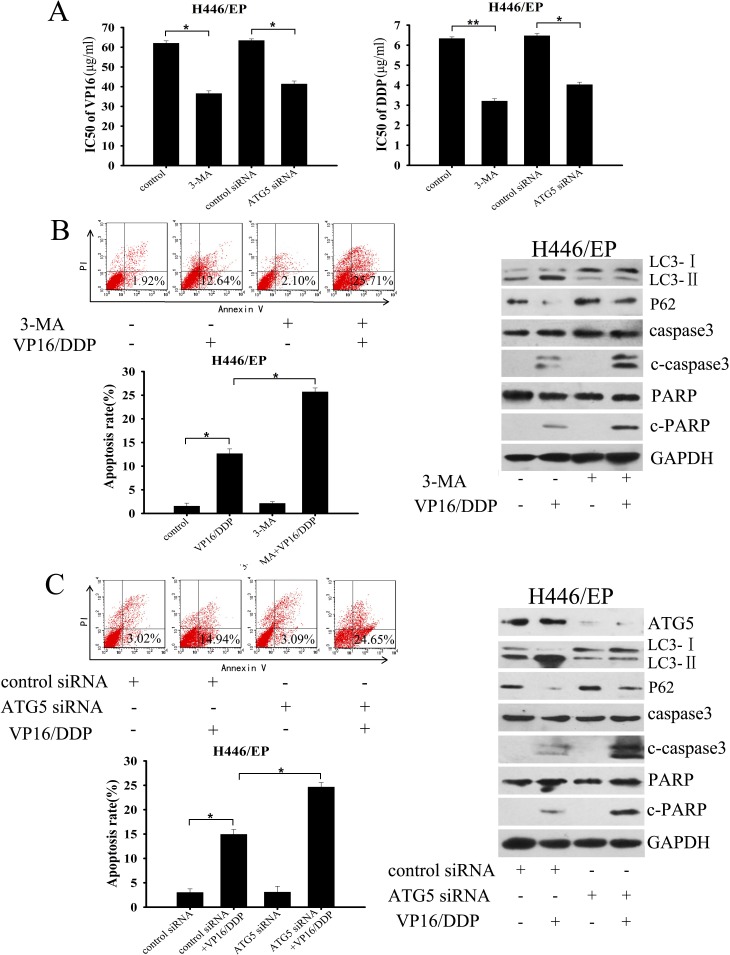
Inhibition of autophagy enhanced sensitivity of H446/EP cells to VP16 and DDP (A) H446/EP cells were pretreated with 3-methlyadenine (3-MA, 5 mM, 2 h) or transiently transfected with either ATG5 siRNA or control siRNA. Cells were then exposed to indicated doses of VP16 or DDP for 48 h. Viability was determined with an MTT assay as described in Materials and Methods. Data are shown as mean ± SD of values from three independent experiments. *P<0.05, **P < 0.01. (B, C) H446/EP cells were treated with VP16–DDP in the presence or absence of (B) 3-MA (5mM, 2 h) or (C) Atg5 siRNA. Autophagy was examined by western blot analysis using specific antibodies against LC3 and p62. Apoptosis was determined by flow cytometric analysis of Annexin-V/PI staining and western blot analysis of cleaved caspase3 (c-caspase3) and cleaved-PARP (c-PARP). Blots were representative of three independent experiments with similar results.

We next explored whether inhibition of autophagy would enhanced the cellular response to chemotherapy. Results from the MTT assay showed that the sensitivity of H446/EP cells to VP16 and DDP was markedly restored after adding 3-methlyadenine (3-MA) or silencing *Atg5* by small-interfering RNA (siRNA) (Fig. 3A). Both 3-MA and *Atg5* siRNA efficiently attenuated activation of autophagy, which led to an enhanced apoptosis rate and marked increases in c-caspase3 and c-PARP, even at low doses of VP16–DDP (Fig. 3B, C). Collectively, all these data validated the concept that chemoresistance in SCLC cells is accompanied by elevated autophagic activity.

### Mir-24-3p blocked autophagy in SCLC cells

**Figure 4:**
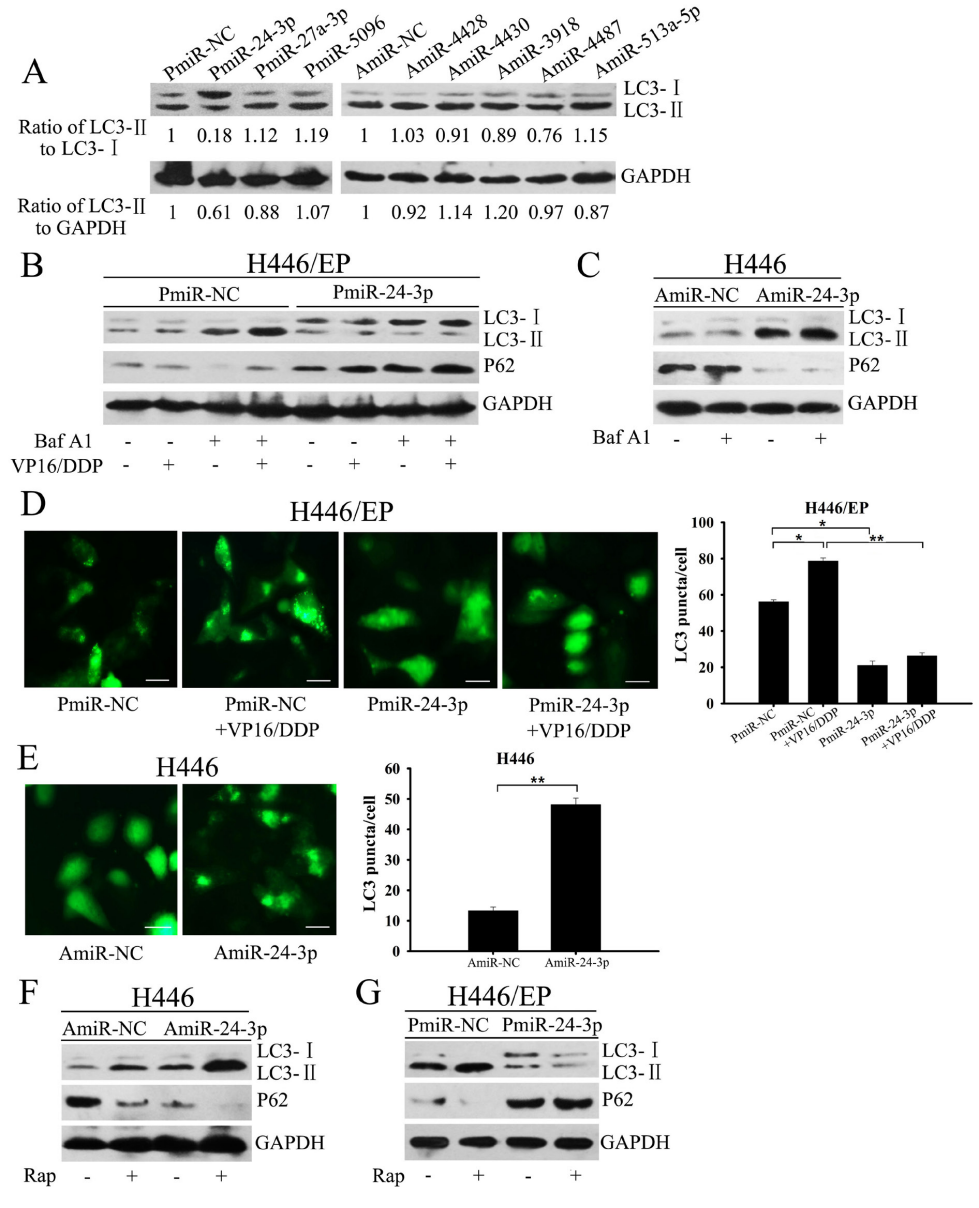
Mir-24-3p blocked autophagy in SCLC cells (A) LC3-II/LC3-I protein was detected by western blot in H446/EP cells transfected with indicated miRNA precursors. Ratios of LC3-II/LC3-I and LC3-II/GAPDH were calculated by gray scale value analysis. (B) H446/EP cells transfected with PmiR-NC or PmiR-24-3p were treated with VP16–DDP, with or without Baf A1 (20 nM, 2 h). (C) H446 cells were transfected with AmiR-NC or AmiR-24-3p, and then exposed to Baf A1 (20 nM, 2 h). Cell lysates were analyzed by western blot for LC3 and P62 (control: GAPDH). (D) H446/EP cells were co-transfected with either control or PmiR-24-3p, and GFP-LC3 plasmid and then treated with VP16–DDP (bar: 50 μm). (E) H446 cells were co-transfected with either control or AmiR-24-3p and GFP-LC3 plasmid (bar: 50 μm). Values are shown as mean ± SD of three independent experiments. *P < 0.05; **P < 0.01. (F) H446 cells transfected with AmiR-24-3p and (G) H446/EP cells transfected with PmiR-24-3p were treated with rapamycin (50 nM, 2 h). Total cell lysates were analyzed by western blot for LC3 and p62. The blots shown are representative of three separate experiments in which similar results were observed.

We hypothesized that these dysregulated miRNAs helped to increase autophagy in H446/EP cells. To verify the hypothesis, we overexpressed downregulated miRNAs or inhibited upregulated miRNAs by transfecting H446/EP cells with miRNA mimics (PmiRNA) or inhibitors (AmiRNA), whose expression patterns were confirmed by qRT-PCR (Supplementary Fig. 1A, B), and then tested autophagic activity. Quantitative assessment of LC3-II/LC3-I and LC3-II/GAPDH ratios served as primary evidence of autophagy induction. Among the dysexpressed miRNAs, miR-24-3p overexpression had the strongest inhibitory effect on autophagy induction, as shown by decreased LC3-II/LC3-I ratio (Fig. 4A). We thus selected miR-24-3p to further explore the influence of miRNAs on autophagy.

H446/EP cells with relatively low miR-24-3p expression were transfected with miR-24-3p mimics (PmiR-24-3P) to upregulate miR-24-3P expression. Forced expression of miR-24-3p led to LC3-I accumulation coupled with diminished LC3-II levels and prevented p62 degradation in fed state and more significantly after VP16–DDP treatment (Fig.4B). As both blockade of autophagosome formation and excessive autophagosome degradation can reduce LC3-II levels, Baf A1 was used to distinguish between these two possibilities. After VP16–DDP treatment, the LC3-II level was further enhanced in Baf A1-pretreated control H446/EP cells, whereas no significant increase was observed in PmiR-24-3p transfected cells. The effect of miR-24-3p on autophagy inhibition was identified by GFP-LC3 fluorescence microscopy, measured as a reduced percentage of punctate GFP^+^ H446/EP cells (Fig.4D). We had opposite results when we silenced miR-24-3p by transfecting a miR-24-3p inhibitor (AmiR-24-3P) into H446 cells. LC3-II expression and punctate GFP^+^ cells were measured after AmiR-24-3p treatment, but were minimally altered in the presence of Baf A1 compared with negative controls (Fig. 4C, E).

To better evaluate the effects of miR-24-3p on the autophagic process, a well-established autophagy inducer, rapamycin (RAP), was applied as a positive control after separate transfections of AmiR-24-3p and PmiR-24-3p into H446 and H446/EP cells, respectively. RAP acts through indirect inhibition of mTORC1, an autophagy-suppressive regulator, followed by autophagy stimulation [20]. Both RAP administration and AmiR-24-3p transfection promoted the conversion of LC3-I to LC3-II compared with the untreated groups in parental H446 cells (Fig. 4F). Notably, co-treatment of AmiR-24-3p and RAP led to an additive effect on the LC3-II expression in H446 cells (Fig. 4F), whereas the LC3-II/LC3-I ratio was markedly weakened by PmiR-24-3p transfection coupled with RAP in H446/EP cells (Fig. 4G). These results indicate that miR-24-3p inhibits autophagy in SCLC cells.

### Mir-24-3p suppressed autophagy by directly targeting *ATG4A*

**Figure 5:**
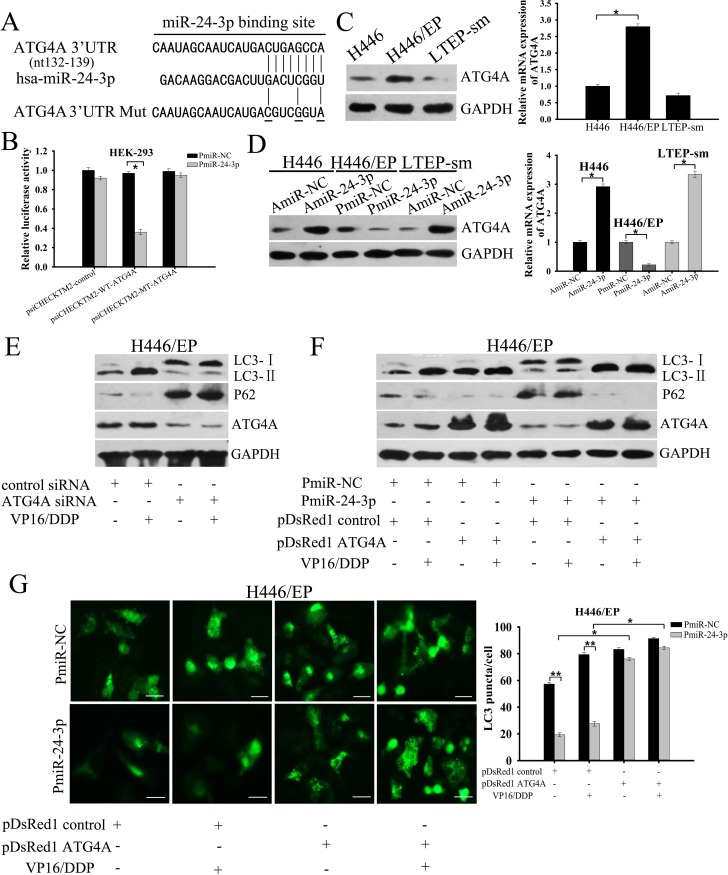
Mir-24-3p suppresses autophagy by directly targeting ATG4A (A) The predicted binding sequence of miR-24-3p within human *ATG4A* 3′UTR. (B) Luciferase activity analysis of *ATG4A* 3′UTR (wild type and mutant constructs) after co-transfection with PmiR-24-3p in HEK-293 cells. (C) Western blot and qRT-PCR validate ATG4A expression in H446 and H446/EP cells. (D) H446 cells were transfected with AmiR-NC or AmiR-24-3p; H446/EP cells were transfected with PmiR-NC or PmiR-24-3p. Western blot and qRT-PCR show ATG4A expression. (E) Western blot shows total cell lysates examined with specific antibodies against LC3, p62 and ATG4A in H446/EP cells transfected with ATG4A siRNA or control siRNA, and then treated with VP16–DDP for 48 h. (F) Western blot for LC3, p62 and ATG4A (control: GAPDH) of lysates of H446/EP cells transfected with PmiR-24-3p, pDsRed1–ATG4A or both, and then exposed to VP16–DDP for 48 h. (G) H446/EP cells were co-transfected with GFP-LC3 plasmids and PmiR-24-3p, pDsRed1–ATG4A, or both combinations (bar: 50 μm). Values are shown as mean ± SD of three independent experiments. *P < 0.05; **P < 0.01.

To elucidate the mechanisms by which miR-24-3p inhibits autophagy, we first searched for autophagy-related targets of miR-24-3p, using miRNA target prediction programs. The MirDB and Targetscan bioinformatics tools helped us find a potential miR-24-3p target sequence in the autophagy gene *ATG4A* 3′-UTR (Supplementary Fig. 2A, B). We used a dual-luciferase reporting system to see whether miR-24-3p affected *ATG4A* by directly targeting this specific complementary sequence in its 3′-UTR region. Co-transfection of miR-24-3p^+^ cells with a wild-type *ATG4A* 3′-UTR reporter construct greatly repressed luciferase activity in both HEK-293 and H446/EP cells, but did not apparently change cells co-transfected with mutant *ATG4A* 3′-UTR reporter construct (Fig. 5A, B; Supplementary Fig. 3). Consistent with the results, ATG4A mRNA and protein expression were increased in H446/EP cells (Fig. 5C). We next checked ATG4A levels in both H446 and H446/EP cells after regulating miR-24-3p expression. PmiR-24-3p led to attenuation of ATG4A mRNA and protein levels in H446/EP cells, but AmiR-24-3p showed the opposite effect on ATG4A expression in H446 cells (Fig. 5D; Supplementary Fig. 1D). Similar to the phenotype induced by miR-24-3p overexpression, *ATG4A* knockdown limited both LC3 lipidation and P62 degradation in H446/EP cells, indicating inhibition of autophagy (Fig. 5E).

To further confirm that miR-24-3p modulates autophagy by directly repressing *ATG4A*, we conducted rescue experiments through ATG4A overexpression. Autophagy blockage induced by PmiR-24-3p returned to control levels after co-transfection of H446/EP cells with cDNA that expressed *ATG4A* (Fig. 5F, G).

To test whether the effect of miR-24-3p on autophagy was cell type-specific, we performed similar analyses in another SCLC cell line, LTEP-sm. ATG4A expression in LTEP-sm cells was slightly lower than in H446 cells (Fig.5C), and miR-24-3p expression was mildly higher (Supplementary Fig. 1C). Consistent with our results in H446 cells, both ATG4A mRNA and protein levels greatly increased in LTEP-sm cells transfected with AmiR-24-3P, but not in cells transfected with control plasmid (Fig.5D; Supplementary Fig. 1D). Administering miR-24-3p led to conversion of LC3-I to LC3-I I, which was reversed by adding a specific siRNA that targets *ATG4A* (Supplementary Fig. 4). These results demonstrated that miR-24-3p directly targeted *ATG4A* to repressively mediate autophagy induction.

### Forced miR-24-3p expression resensitized SCLC cells to chemotherapy by blocking autophagy

**Figure 6:**
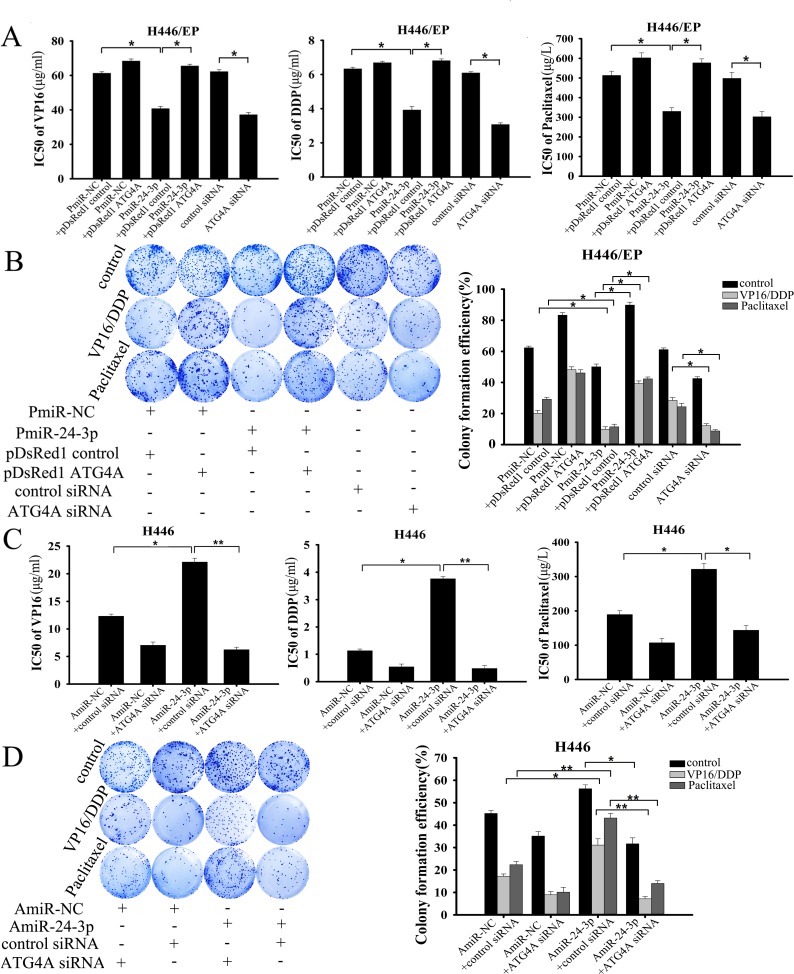
MiR-24-3p altered the chemosensitivity of SCLC cells by targeting ATG4A (A, B) H446/EP cells were transfected with PmiR-24-3p, pDsRed1–ATG4A, both of them or ATG4A siRNA, followed by treatment with indicated concentrations of VP16, DDP or paclitaxel for 48 h. (A) MTT assay shows cell viability; (B) colony formation assay shows cell proliferation. (C, D) H446 cells were transfected with AmiR-24-3p, ATG4A siRNA or both, and then treated with indicated concentrations of VP16, DDP or paclitaxel for 48 h. (C) MTT assay shows cell viability; (D) colony formation assay shows cell proliferation. Results show three identical experiments (bars: mean ± SD; *P < 0.05; **P < 0.01).

**Figure 7:**
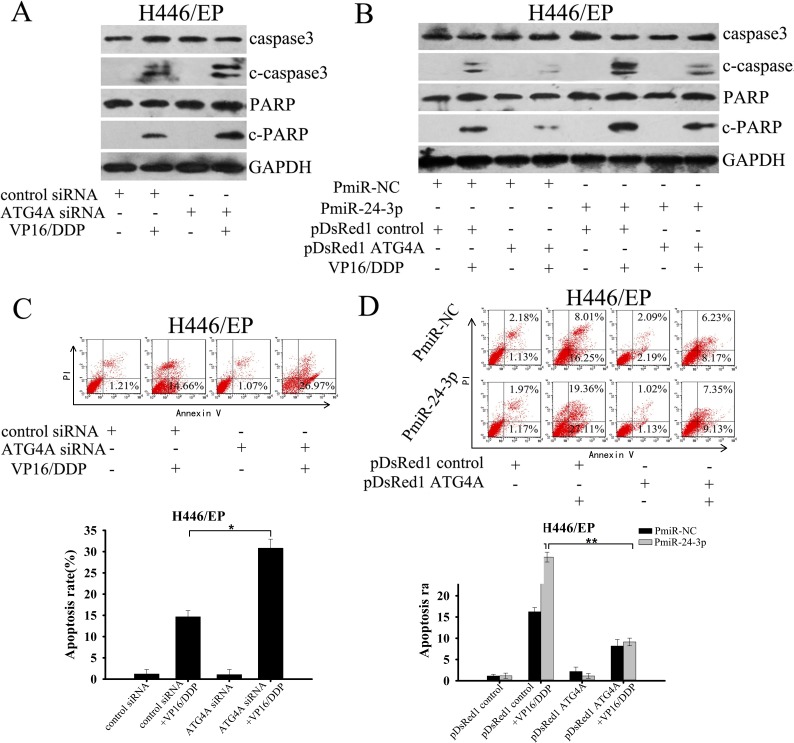
MiR-24-3p altered the sensitivity of SCLC cells to apoptosis through by ATG4A (A, C) H446/EP cells were transfected with ATG4 siRNA before incubation with VP16–DDP for 48 h. Apoptosis was analyzed by (A) western blot for c-caspase3 c-PARP; and (C) flow cytometry with Annexin-V staining. (B, D) H446/EP cells were transfected with PmiR-24-3p, pDsRed1–ATG4A or both before treatment with VP16–DDP. Apoptosis was analyzed by (B) western blot for c-caspase-3 c-PARP; and (D) flow cytometry with Annexin-V staining. Results show three identical experiments (bars: mean ± SD; *P < 0.05; **P < 0.01).

We next verified that miR-24-3p inhibited SCLC cell proliferation and survival in response to VP16–DDP by attenuating the protective effect of autophagy through suppressing ATG4A. Either restoration of miR-24-3p or silencing *ATG4A* effectively increased cytotoxicity and decreased proliferation in H446/EP cells, as shown by MTT and colony formation assays, respectively (Fig. 6A, B). By contrast, introduction of exogenous ATG4A promoted drug resistance in H446/EP cells that overexpressed miR-24-3p. Moreover, similar results were obtained in H446/EP cells transfected with PmiR-24-3p or siATG4A following treatment with another cytotoxic agent, paclitaxel. C-caspase-3 and c-PARP expression were simultaneously enhanced by VP16–DDP treatment, in both miR-24-3p-overexpressing and *ATG4A*-silenced H446/EP cells, which implies a restored sensitivity to apoptosis (Fig. 7A, B), which was confirmed by flow cytometry (Fig. 7C, D). However, reduced cell viability and colony formation ability induced by supplementary miR-24-3p was abrogated by ATG4A overexpression (Fig. 6A, B). Furthermore, the sensitivity of H446 and LTEP-sm cells toVP16, DDP or paclitaxel was greatly reduced when any of the drugs were combined with AmiR-24-3p. Nevertheless, suppression of ATG4A restored the cytotoxic effect of AmiR-24-3p in response to chemotherapeutics (Fig. 6C; Supplementary Fig.5A). Similarly, miR-24-3p downregulation promoted colony-formation of these cells under drug treatment, but was reversed by *ATG4A* knockdown (Fig. 6D; Supplementary Fig. 5B).

## DISCUSSION

As far as we know, the present study is the first to report that miR-24-3p suppresses the endogenous autophagy process by directly downregulating ATG4A expression in SCLC cells. Inhibition of autophagy by induced overexpression of miR-24-3p helps resensitize SCLC cells to VP16–DDP combined therapy.

Autophagy is a conserved pro-survival response to chemotherapy to maintain cellular homeostasis, which in turn conduces chemoresistance development in several cancer types [21-23]. Currently, inhibitors of autophagy that sensitize chemoresistant cells to anti-cancer therapy are being investigated in clinical trials [24]. Modulation of autophagy by miRNAs is a quite novel and potentially effective strategy to resensitize cancer cells. Although several miRNAs have so far been reported to regulate autophagic process by targeting autophagy-related genes in diverse cancers, such as hepatocellular carcinoma, leukemia and breast cancer [25-27], few investigations verified miRNAs to directly affect regulation of autophagy in SCLC cells. In this study, we were interested in the role of miRNA in modulating chemoresistance of SCLC cells through autophagy activity. We first confirmed that VP16–DDP-resistant H446 cells showed higher basal level of autophagy than did their parental H446 cells; and then, using microarray analysis, we identified a set of specific differentially expressed miRNAs associated with VP16–DDP resistance. Some of these differential expressed miRNAs are reported to closely correlate with tumor behavior. For example, miR-27a-3p and miR-24-3p synergistically promoted glioma cells proliferation by directly targeting MXL1 [28], whereas overexpression of miR-4430 is related to distant metastasis in salivary adenoid cystic carcinoma cells[29].

However, in our study, of all these dysregulated miRNAs, only induced expression of miR-24-3p into H446/EP cells blockaded autophagy activity and reduced cell viability with VP16–DDP treatment. Overexpression of miR-24-3p attenuated GFP-LC3 dot formation, conversion of LC3-I to LC3-II and P62 degradation. These results still held true even after using an autophagy inducer (rapamycin). In contrast, silence of endogenous miR-24-3p activated the autophagy response in H446 cells, which was further amplified when coupled with rapamycin treatment. Collectively, these data indicate the importance of miR-24-3p in autophagy regulation.

Bioinformatics predictions and a luciferase reporter assay identified the autophagy-related gene *ATG4A* as a direct functional target of miR-24-3p, and further found ATG4A to downregulate in H446/EP cells with miR-24-3p overexpression. Of four mammalian ATG4 family members, ATG4B reportedly shows the most active and broadest proteolysis on human ATG8 orthologs, followed by ATG4A, and other family members [15]. However, tissue- or cell-specific roles for ATG4 might vary in mammalian systems and complicate interpretation of their individual functions. *ATG4C*-knockout mice exhibited decreased autophagic activity upon starvation, but had weak autophagy defect under normal conditions [17]. *ATG4C* was also regarded as a direct target of miR-376b in human hepatocarcinoma cell lines, in which it promotes LC3 maturation [30]. Another counter-example can be seen in the mammosphere formation in breast cancer cells. Overexpression of ATG4A, but not ATG4B, in mammospheres reportedly contributes to breast cancer stem cell maintenance, owing to its importance in autophagosomal maturation [31]. During early erythroid differentiation (when autophagy was activated) expression of ATG4A and ATG4D was markedly increased, whereas ATG4B showed minimal change [32]. These results show the potential role of ATG4A in an efficient autophagic process.

In our study, we detected no prominent increase of the lipidated LC3-II form after miR-24-3p upregulation and *ATG4A* silence, but did observe accumulation of the nonlipidated LC3-I form accompanied by an enhanced expression of p62 protein, which suggests interruption of LC3 maturation at the lipidation stage. This is consistent with the early idea that ATG4B rather than ATG4A is responsible for LC3 protein delipidation [33]. MiR-24-3p overexpression led to dramatic decreases in ATG4A mRNA and protein levels, but reintroduction of ATG4A in the presence of miR-24-3p reversed autophagy suppression. The effect of miR-24-3p on *ATG4A* occurred directly through its 3′UTR region, and introduction of mutations to this sequence efficiently abolished the miRNA's effect. In addition, we attempted to investigate whether miR-24-3p could modulate the expression of other ATG4 proteins. Unfortunately, we observed no obvious difference on the baseline levels of these ATG4 proteins (Supplementary Fig.6A). All three of ATG4 mRNA and protein expression were minimally altered after either transfection of AmiR-24-3p into H446 and LTEP-sm cells or transfection of PmiR-24-3p into H446/EP cells (Supplementary Fig.6B, C).

Gene amplification and overexpression of miR-24 are reported in several cancer types, including oral squamous cell carcinoma, hepatocellular carcinoma and glioma; and correlate with tumorigenesis, progression, and aggressiveness [34-36]. Moreover, miR-24 has been associated with key regulation of many genes involved in apoptosis induction. MiR-24-3p overexpression helps reverse resistance to apoptosis by downregulating expression of two apoptosis blockers, X-linked inhibitor of apoptosis protein and lysophosphatidic acid acyltransferase-β [37, 38]. To our knowledge, however, the role of miR-24-3p in regulating autophagy remained undefined. Our study of the contribution of autophagy-related effects of miR-24-3p could provide valuable information about the role of this miRNA under physiological conditions.

**Figure 8:**
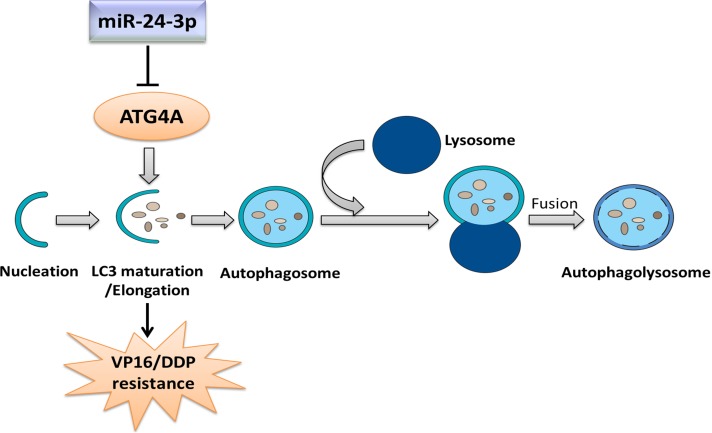
Mechanism through which miR-24-3p modulates VP16–DDP resistance by regulating autophagy MiR-24-3p attenuates autophagy by targeting the key autophagy protein ATG4A, thus preventing LC3 maturation/membrane elongation autophagy stage.

Strikingly, both miR-24-3p overexpression and *ATG4A* knockdown greatly reduced cell viability, inhibited cell proliferation and promoted apoptosis; these effects were even more pronounced when VP16−DDP-resistant cells were treated with VP16–DDP or paclitaxel alone. However, these results could be reversed after restoring ATG4 expression. Our findings strongly indicate that autophagy inhibition through miR-24-3p-mediated targeting of *ATG4A* in SCLC cells cuts the cells' survival response and enhances chemotherapy cytotoxicity (Fig. 8). Further *in vivo* studies could uncover beneficial effects of autophagy suppression by miR-24-3p. In fact, autophagy inhibition combined with cancer therapy has been proposed as an approach to potentiate tumor cell death. Some dysexpressed miRNAs have been shown to affect sensitivity to radio-or chemotherapy by regulating autophagy in several types of cancer cells [25-27]. However, the details of mechanisms and intracellular signaling pathways through which miRNAs exert their function, especially on autophagy regulation, are unclear. Further intensive research is needed to elucidate relationships between aberrant miRNA expression and autophagy abnormalities to develop use of miRNAs into a tool of improved cancer therapy [39].

In summary, we report here, for the first time, that miR-24-3p is a novel regulator of autophagy in SCLC cells. MiR-24-3p mediated autophagy regulation by targeting *ATG4A* and thereby contributed to VP16–DDP resistance. Inhibition of autophagy by elevation of miR-24-3p might provide a useful strategy for combatting chemoresistance in SCLC cells.

## MATERIALS AND METHODS

### Cell lines and reagents

Human small-cell lung carcinoma H446 and LTEP-sm cells were purchased from the Tumor Cell Bank of the Chinese Academy of Medical Science (Shanghai, China) and cultured in RPMI 1640 medium containing 10% fetal bovine serum and ampicillin and streptomycin at 37ºC in a humidified atmosphere of 95% air and 5% CO_2_. VP16–DDP-resistant H446 cells (H446/EP) were established and preserved in a final concentration of 1.5μg/ml VP16 and concentration of 1.25μg/ml DDP. Antibodies against LC3, P62, caspase-3, activated (cleaved) caspase-3 (c-caspase-3), PARP, cleaved PARP (c-PARP), Atg5 and GAPDH came from Cell Signaling Technology, (MA, USA). Anti-ATG4A, Anti-ATG4B, Anti-ATG4C and Anti-ATG4D antibodies came from Abcam. Bafilomycin A1 and 3-methyladenine (3-MA) came from Sigma Aldrich (St. Louis, MO).

### cDNA constructs, siRNA and transfection

The GFP-tagged LC3 cDNA expression construct was a gift from Dr. Noboru Mizushima (Tokyo Medical and Dental University, Tokyo, Japan). Both miR-24-3p inhibitor (AmiR-24-3p) and miR-24-3p precursor (PmiR-24-3p) were provided by Applied Biosystem (Life Technologies). *ATG5* siRNA, *ATG4A* siRNA, pDsRed1/Atg4A were purchased from GenePharma (Shanghai, China). Cells were transfected by using Lipofectamine 2000 (Invitrogen, USA), according to the manufacturer's protocol.

### Cell viability

Cells were seeded into 96-well plates (3×10^3^ cells/well) directly or 24 h after transfection. After treatment with indicated drug combinations for 48 h, cell viability was assessed via 3-(4,5-dimethylthiazol-2-yl)-2,5-diphenyl-trtrazolium bromide (MTT) assay as described previously [40].

### Colony formation assay

After 48h transfection, cells were exposed to various treatments, and then seed into 6-well plates. Cells were allowed to adhere and grow for between 10 to 14 d. To visualize colonies, cells were fixed with methanol and stained with 0.1% crystal violet. Colonies with ≥50 cells were manually counted under a dissection microscope.

### Western blot analysis

Cells were lysed in RIPA buffer; protein concentrations were determined with a BCA kit (Pierce, Rockford, IL, USA). Equal amount of cell lysates were subjected to SDS-PAGE, transferred onto nitrocellulose members, and analyzed as described previously [41].

### Real-time quantitative PCR (qRT-PCR) analysis

Total RNAs were extracted from cells with Trizol (Invitrogen) Reagent following the manufacturer's protocol. For miRNA detection, SYBR PrimeScript™ miRNA RT-PCR kit (Takara, Japan, Cat. No. RR716) was used according to manufacturer's instructions. The conditions for PCR reactions were: 95°Cfor 30 s followed by 40 cycles of 95°C for 5 s and 60°C for 30 s, using a StepOnePlus™ thermal cycler. MiRNA expression levels were normalized to *U6* RNA. SYBR Premix Ex TaqTM (Takara, Japan, Cat. No. RR420A) was also used to detect mRNA. SYBR Green quantitative PCR amplifications were performed on a StepOnePlus™ thermal cycler, in 25μl volumes containing 12.5μl of 2×SYBR Green PCR Master Mix. The thermal profile for real-time PCR was: 95°C for 30 s; followed by 40 cycles of 95°C for 5 s, 60°C for 30 s and 70°C for 30 s. Relative mRNA expression was normalized to *GAPDH* using the comparative ΔΔC_T_ method; values are expressed as 2^−ΔΔCT^. Primer sequences are listed in Supplementary Table 2.

### Apoptosis assay

We measured apoptosis using an Annexin-V-fluorescein isothiocyanate apoptosis detection kit (Oncogene Research Products, Boston, MA) that quantitatively measures percentages of early apoptotic cells via flow cytometry. Western blot analyses for c-PARP and c-caspase3 after various treatments were also performed.

### Dual luciferase reporter assay

The 3′-UTR-fragments of *ATG4A* containing miR-24-3p targeting sequence were cloned into the psiCHECK^TM^-2 dual luciferase reporter plasmid at the 3′ end of the of *R. reniformis* luciferase coding sequence. For the reporter assay, cells were cultured to approximately 80% confluence in a 6-well plate, and then co-transfected with either psiCHECKTM2-WT-ATG4A-3′UTR (wild type) or psiCHECKTM2-MT-ATG4A-3′UTR (mutant) vector and control miRNA (PmiR-NC) or miR-24-3p mimic (PmiR-24-3p). At 48 h after transfection, firefly and *Renilla* luciferase activities were measured and normalized using the Dual-Luciferase Reporter Assay.

### GFP-LC3 analysis

Twenty-four hours after GFP-LC3 transfection, cells were fixed in 3.7% formaldehyde for 20 min, washed with PBS, mounted in glycerol in PBS and inspected using a fluorescence microscope. Both H446 and H446/EP cells with ≥10 GFP-LC3 dots were considered positive. The number of cells with punctate fluorescence was counted in 10 different fields under a fluorescent microscope. At least 150 GFP^+^ cells per condition were analyzed; results were expressed as percentages of GFP-LC3 dot^+^ cells vs. total number of GFP^+^ cells.

### Transmission electron microscopy

Cells were fixed with a solution containing 3% glutaraldehyde plus 2% paraformaldehyde in 0.1 mol/L phosphate buffer (pH 7.4), followed by 1% OsO_4_. After dehydration, thin sections were stained with uranyl acetate and lead citrate for observation under a JEM 1011CX electron microscope (JEOL, USA, Inc.). Digital images were obtained using an Advanced Microscopy Techniques imaging system.

### Statistical analysis

Data are expressed as mean ± SD of ≥ 3 separate experiments. SPSS17.0 software was used for statistical analysis. Multiple group comparisons were analyzed with one-way ANOVA; 2-group comparisons were performed with Student's *t* test. *P* < 0.05 was considered statistically significant.

## SUPPLEMENTARY MATERIAL FIGURES AND TABLES



## ABBREVIATIONS

3′UTR: 3′-untranslated region
3-MA: 3-methlyadenine
ATG4A: autophagy-associated gene 4A
Baf A1: Bafilomycin A1
DDP: cisplatin
LC3: microtubule-associated protein 1 light chain 3
miRNAs: microRNAs
qRT-PCR: real-time quantitative RT-PCR
RAP: rapamycin
SCLC: small-cell lung cancer
siRNA: small-interfering RNA
VP16: etoposide
VP16.DDP: combined VP16 and DDP treatment
