# Supplementary material for: Mir-24-3p downregulation contributes to VP16–DDP resistance in small-cell lung cancer by targeting ATG4A
Source: Oncotarget. 2014 Nov 16;6(1):317–31. doi: 10.18632/oncotarget.2787 (PMC4381597; doi:10.18632/oncotarget.2787)
Supplement: Supplementary file 1 [file oncotarget-06-317-s001.pdf]

Mir-24-3p downregulation contributes to VP16–DDP resistance in small-cell lung cancer by targeting *ATG4A*

Supplementary Material

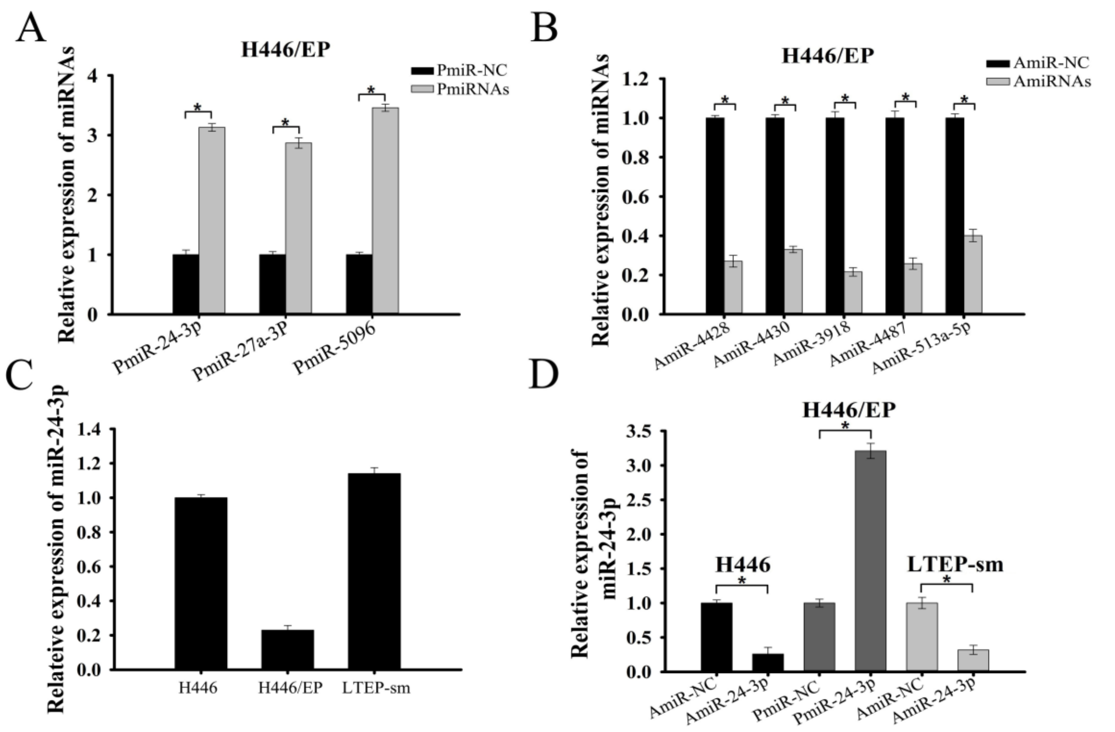

Supplementary Figure 1: Effect of miRNAs precursors on SCLC cells. H446/EP cells were separately transfected with one of **(A)** 3 PmiRNAs or **(B)** 5 AmiRNAs. Relative expressions of miRNAs were determined by qRT-PCR. **(C)** qRT-PCR was performed to detect the relative expression of miR-24-3p in H446, H446/EP and LTEP-sm cells. **(D)** After transfection of AmiR-24-3p in to H446 and LTEP-sm cells or transfection of PmiR-24-3p in to H446/EP cells, relative expressions of miRNAs were verified by qRT-PCR. Values are reported as mean ± SD of three independent experiments. *$P < 0.05$; **$P < 0.01$.

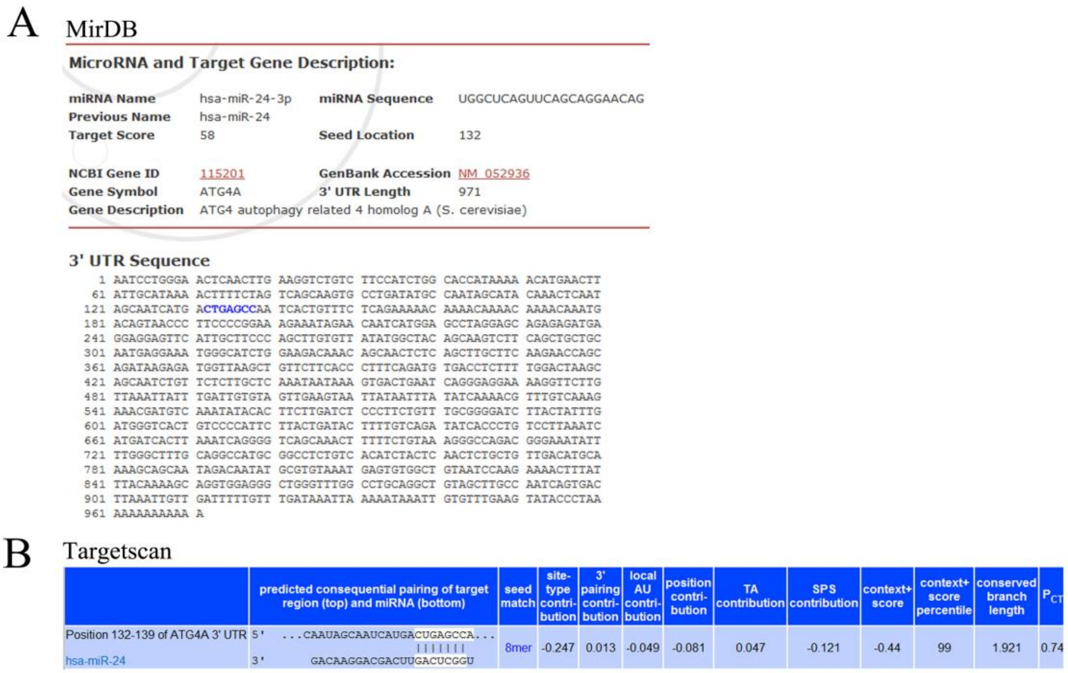

Supplementary Figure 2: Prediction for miR-24-3p regulated targets. Base pairing complements were analyzed with **(A)** MirDB and **(B)** Targetscan bioinformatics tools.

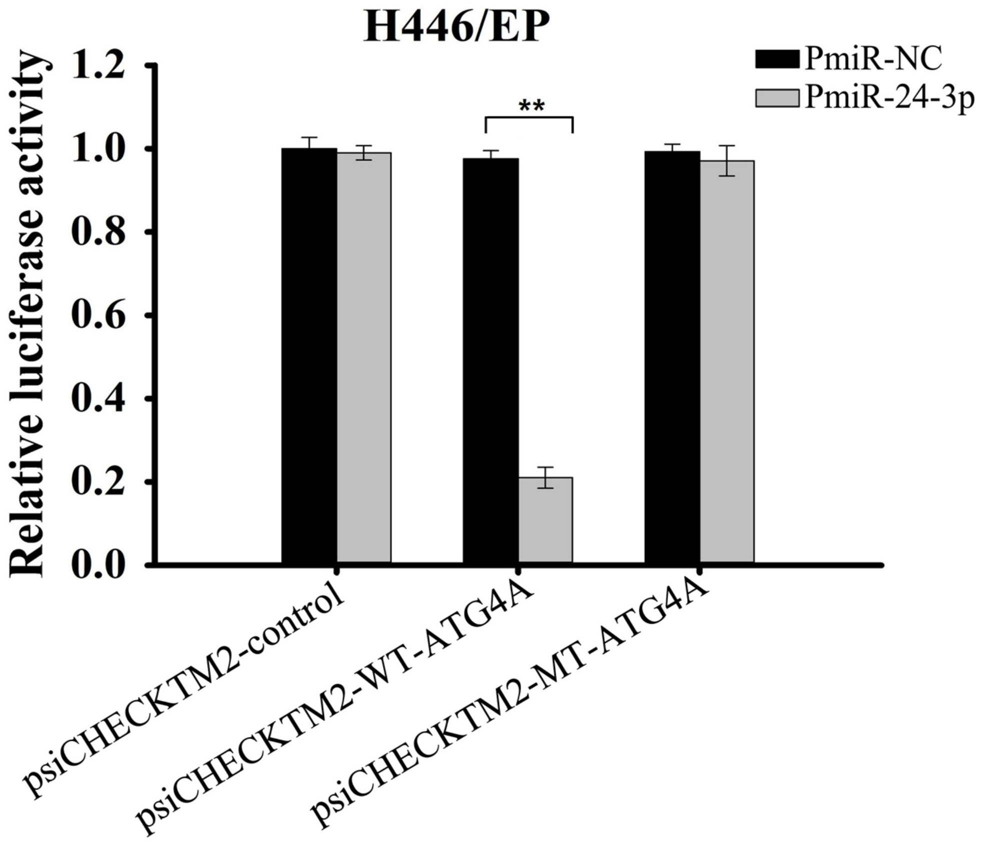

Supplementary Figure 3: ATG4A is a direct target of miR-24-3p. Luciferase activity analysis of ATG4A 3′UTR (wild type and mutant constructs) after co-transfection with PmiR-24-3p in H446/EP cells. For each experiment, data were normalized to luciferase activity detected in cells transfected with NC. $^{**}P < 0.01$.

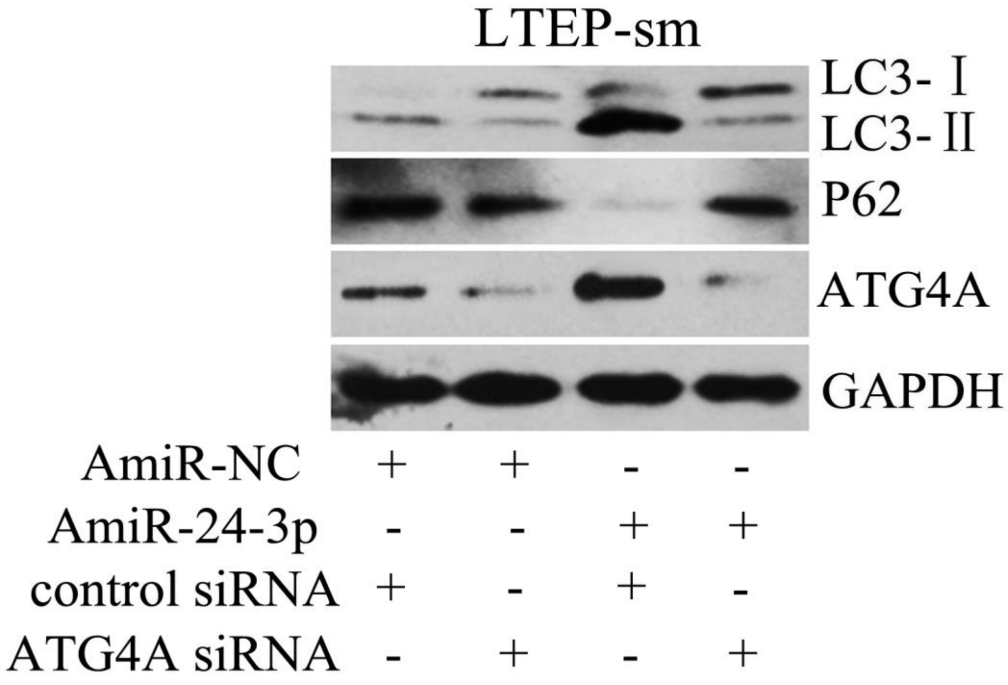

Supplementary Figure 4: Downregulated miR-24-3p promotes autophagy activity of LTEP-sm cells. LTEP-sm cells were transfected with AmiR-24-3p, ATG4A siRNA or both. Whole cell lysates were analyzed by western blot for LC3, P62 and ATG4A. GAPDH was used as a loading control. Results are from ≥ 3 independent experiments.

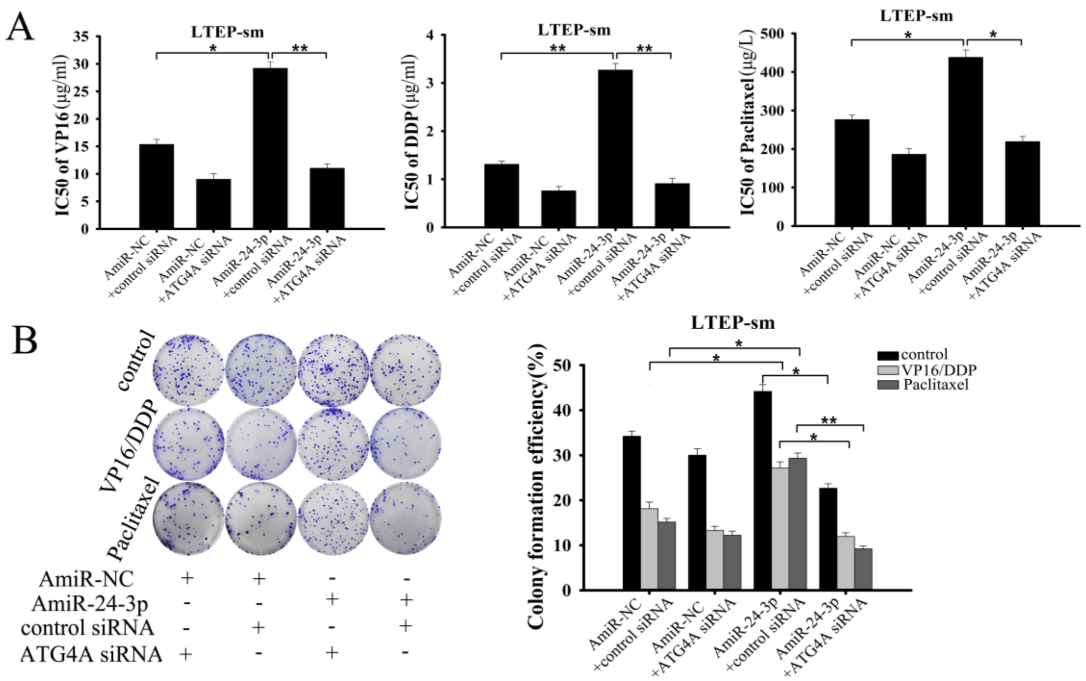

Supplementary Figure 5: Downregulated miR-24-3p increases resistance of LTEP-sm cells to chemotherapy. (A, B) H446 cells were transfected with AmiR-24-3p, ATG4A siRNA or both, and then treated with indicated concentrations of VP16, DDP or paclitaxel for 48 h. **(A)** MTT assay shows cell viability; **(B)** colony formation assay shows cell proliferation. Results show three identical experiments (bars: mean ± SD; *$P < 0.05$; **$P < 0.01$).

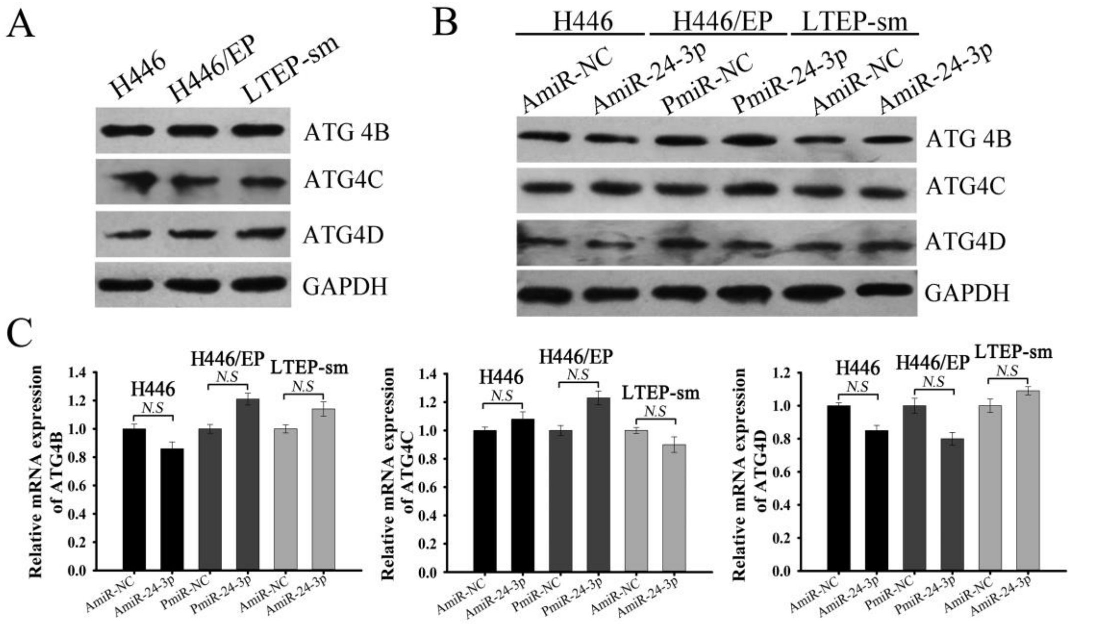

Supplementary Figure 6: MiR-24-3p had no effect on the other ATG4 expressions in SCLC cells. (A) Western blot for ATG4B, ATG4C and ATG4D (control: GAPDH) in H446 cells, H446/EP and LTEP-sm cells. **(B, C)** After transfection of AmiR-24-3p in to H446 and LTEP-sm cells or transfection of PmiR-24-3p in to H446/EP cells, the protein and mRNA expressions of ATG4B, ATG4C and ATG4D were analyzed by **(B)** western blot and **(C)** qRT-PCR, respectively.

Supplementary Table 1: Eight differentially expressed miRNAs. MiRNAs that were differentially expressed more than 2.5 fold in H446/EP cells compared to H446 cells.

Name	Intensity in H446	Intensity in H446/EP	Ratio(H446/EP vs. H446)
hsa-miR-24-3p	1634.3892	643.4574	0.39370
hsa-miR-27a-3p	3800.7899	1170.4350	0.30795
hsa-miR-5096	264.7686	100.5109	0.37962
hsa-miR-4428	595.1107	1810.1350	3.04168
hsa-miR-4430	392.2039	1154.4460	2.94354
hsa-miR-3918	217.7536	578.6420	2.65733
hsa-miR-4487	2230.7371	6945.5829	3.11358
hsa-miR-513a-5p	655.7352	1791.3479	2.73185

Supplementary Table 2: Primers used in this study.

Name	Primer Sequence
**Primer for qRT-PCR**	
miR-24-3p-F	5’-TGGCTCAGTTCAGCAG-3’
miR-27a-3p	5’-TTCACAGTGGCTAAGTTC-3’
miR-5096-F	5’-AAGTAGAGGTGGGGTTT-3’
MiR-4428-F	5’-TTGGCAGGTGCCATGT-3’
miR-4430-F	5’-AATATGTGAGGCTGGAGT-3’
miR-3918-F	5’-AACTTTGTTCGTTCGGC-3’
miR-4487-F	5’-TTTACTGTCCTTCAGCCA-3’
miR-513a-5p-F	5’-TTCACAGGGAGGTGTC-3’
U6-F	5’-GCTTCGGCAGCACATATACTAAAAT-3’
U6-R	5’-CGCTTCACGAATTTGCGTGTCAT-3’
ATG4A-F	5’-TTGGCCCAGGATGACAGCTG-3’
ATG4A-R	5’-AGGGCCCGTTCCACCAATTG-3’
ATG4B-F	5’-TGAGTCTTGTGGTGTGTGGT-3’
ATG4B-R	5’-TACTTTCCCAGGACAGGCAG-3’
ATG4C-F	5’-GTTACCTGCAGAGTCGGGAT-3’
ATG4C-R	5’-GGCCAGTTCTCAATGTGCAG-3’
ATG4D-F	5’-GTCCATGAACTCAGTGTCGC-3’
ATG4D-R	5’-GAACTTGTCCACTTCGTCCG-3’
GAPDH-F	5’-GGGAGCCAAAAGGGTCATCATCTC-3’
GAPDH-R	5’-CCATGCCAGTGAGCTTCCCGTTC-3’

Abbreviations: F, forward primer; R, reverse primer.
